# Charge-transfer biexciton annihilation in a donor–acceptor co-crystal yields high-energy long-lived charge carriers†

**DOI:** 10.1039/d0sc03301d

**Published:** 2020-08-13

**Authors:** Itai Schlesinger, Natalia E. Powers-Riggs, Jenna L. Logsdon, Yue Qi, Stephen A. Miller, Roel Tempelaar, Ryan M. Young, Michael R. Wasielewski

**Affiliations:** Department of Chemistry and Institute for Sustainability and Energy at Northwestern, Northwestern University 2145 Sheridan Road Evanston Illinois 60208-3113 USA m-wasielewski@northwestern.edu

## Abstract

Organic donor–acceptor (D–A) co-crystals have attracted much interest due to their important optical and electronic properties. Co-crystals having ⋯DADA⋯ π-stacked morphologies are especially interesting because photoexcitation produces a charge-transfer (CT) exciton, D˙^+^–A˙^−^, between adjacent D–A molecules. Although several studies have reported on the steady-state optical properties of this type of CT exciton, very few have measured the dynamics of its formation and decay in a single D–A co-crystal. We have co-crystallized a *peri*-xanthenoxanthene (**PXX**) donor with a *N*,*N*-bis(3-pentyl)-2,5,8,11-tetraphenylperylene-3,4:9,10-bis(dicarboximide) (**Ph4PDI**) acceptor to give an orthorhombic **PXX**–**Ph4PDI** ⋯DADA⋯ π-stacked co-crystal with a CT transition dipole moment that is perpendicular to the transition moments for S_*n*_ ← S_0_ excitation of **PXX** and **Ph4PDI**. Using polarized, broadband, femtosecond pump–probe microscopy, we have determined that selective photoexcitation of **Ph4PDI** in the single co-crystal results in CT exciton formation within the 300 fs instrument response time. At early times (0.3 ≤ *t* ≤ 500 ps), the CT excitons decay with a *t*^−1/2^ dependence, which is attributed to CT biexciton annihilation within the one-dimensional ⋯DADA⋯ π-stacks producing high-energy, long-lived (>8 ns) electron–hole pairs in the crystal. These energetic charge carriers may prove useful in applications ranging from photovoltaics and opto-electronics to photocatalysis.

## Abstract 2

Femtosecond transient absorption microscopy of donor–acceptor single co-crystals shows that photogenerated charge transfer excitons in one-dimensional donor–acceptor π stacks annihilate to produce high-energy, long-lived electrons and holes.

## Graphical abstract

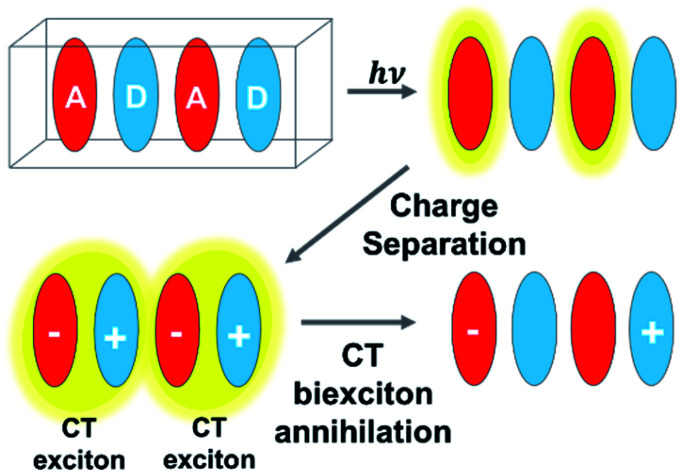

## Introduction

Organic donor–acceptor (D–A) co-crystals have been shown to possess many interesting properties, among them: semiconducting behavior with appreciable ambipolar charge transport capabilities,^1–8^ tunable emission spectra,^5,9–13^ and ferroelectricity.^5,9,14^ These structures are also believed to be good candidates for organic field-effect transistors,^15,16^ organic photovoltaics,^17–19^ photonic logic,^20,21^ and near-IR photothermal imaging.^22^ An important feature of co-crystals having a ⋯DADA⋯ π-stacked morphology is their ability to form charge transfer (CT) excitons within D–A pairs.

Early work of Haarer and co-workers showed that photoexcitation of anthracene and phenanthrene co-crystals with pyromellitic dianhydride (PMDA) initially produces a singlet CT D˙^+^–A˙^−^ exciton that can intersystem cross to give the corresponding triplet CT exciton.^23–25^ The CT exciton hopping rates along the one-dimensional ⋯DADA⋯ π-stacks within the co-crystal depend both on temperature and spin multiplicity with exciton trapping occurring at low temperatures and hopping rates being slower for singlet relative to triplet CT excitons.^26–29^ Several studies characterizing the CT ground state optical absorption as well as the steady-state and time-resolved photoluminescence of D–A co-crystals have been reported.^2,11,12,20,21,30,31^ Also, studies using ultrafast transient diffuse reflectance spectroscopy have been reported on a variety of co-crystal powders, including phenathrene^32^ and methylated benzenes^33,34^ co-crystallized with PMDA, and aromatic donors co-crystallized with tetracyanobenzene^35,36^ and tetracyanoquinodimethane^37^ acceptors. In addition, there have been several studies of exciton transport in polyacene single crystals of interest to singlet fission.^38–40^ However, studies that employ transient optical absorption measurements to study CT exciton dynamics in single D–A co-crystals are relatively rare. In one of the first examples, Port and co-workers reported on the ultrafast CT exciton dynamics in single crystals of anthracene and PMDA, where they observed transient spectral features that they attributed to a Stark effect of the CT exciton on the absorption spectra of nearby molecules.^41,42^

**Fig. 1:**
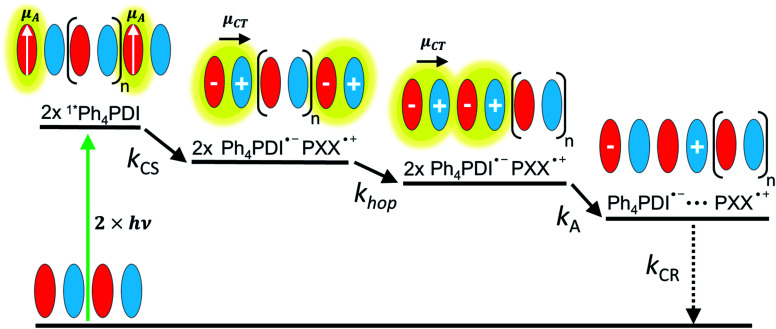
CT biexciton annihilation model. Following photoexcitation, two photons create two singlet Frenkel excitons on **Ph4PDI** acceptor molecules, which then rapidly charge separate to create two CT excitons with a rate constant of *k*_CS_. The transition dipole moment of **Ph4PDI**, *μ*_A_, is perpendicular to the CT exciton transition dipole moment, *μ*_CT_. CT exciton migration with a site-to-site rate constant, *k*_hop_, and collision result in CT biexciton annihilation, which produces spatially separated electron–holes pairs with a rate constant *k*_A_. Alternatively, the CT biexciton can be produced on adjacent D–A pairs resulting in annihilation. The electron–hole pair recombination rate is *k*_CR_.

Given the low dielectric constants of organic solids, the dissociation of CT excitons into free charge carriers is often limited by their inability to overcome the Coulomb barrier necessary to separate the charges. A similar problem is encountered in bulk heterojunction organic photovoltaics, where charges need to migrate efficiently to the anode and cathode for optimal performance.^43–45^ Here, we examine CT exciton hopping (tunneling) and CT biexciton annihilation in one-dimensional ⋯DADA⋯ π-stacks in a single D–A co-crystal as a mechanism by which free charge carriers can be created (Fig. 1). In contrast to Frenkel biexciton annihilation, this mechanism is underexplored, and has been observed previously only in a covalently linked D_1_–A_1_–D_2_–A_2_ molecule in solution.^46^

In this work, we co-crystallized *peri*-xanthenoxanthene (**PXX**) with *N*,*N*-bis(3-pentyl)-2,5,8,11-tetraphenylperylene-3,4:9,10-bis(dicarboximide) (**Ph4PDI**) to give an orthorhombic **PXX**–**Ph4PDI** ⋯DADA⋯ π-stacked co-crystal. Both isolated molecules have S_1_ ← S_0_ transition dipole moments that lie in their core plane and point along their long molecular axes.^47,48^ When co-crystallized, the molecules form one-dimensional stacks and orient themselves such that their transition dipole moments are almost parallel to each other, and both lie in a plane that is exactly perpendicular to the π–π stacking direction. In a ⋯DADA⋯ π-stacked co-crystal, the donor and acceptor orbitals can mix and form delocalized crystal orbitals or bands, depending on the degree of D–A electronic coupling and energy differences. When the coupling is sufficiently strong, the oscillator strength of the direct transition between the ground state to the CT exciton is significant and the resulting CT absorption band is bright. Importantly, the CT exciton in a ⋯DADA⋯ π-stacked co-crystal has a distinct transition dipole moment along the π-stacking direction, which can be independently probed in spectroscopic measurements of an aligned co-crystal. The CT absorption band in the **PXX**–**Ph4PDI** co-crystal is polarized along the long crystal axis, *i.e.* the π–π stacking direction (Fig. S1†), while other spectral components are polarized perpendicular to this axis, and parallel to the donor and acceptor S_1_ ← S_0_ transition dipole moments (Fig. 1).

To probe the ultrafast dynamics of CT excitons in D–A co-crystals, it is most informative to conduct femtosecond time-resolved spectroscopic measurements over a broad spectral range, taking advantage of the anisotropic absorption changes that can be directly related to the orientation of the donor and acceptor molecules relative to the crystal axes. This makes it possible to distinguish spectral changes attributable to the CT exciton from those resulting from contributions from other competing processes. Since the co-crystals are usually only a few micrometers in size, regular femtosecond transient absorption (fsTA) experiments having several hundred micrometer pump and probe beam spot sizes inherently probe a heterogenous collection of crystal orientations. This leads to averaging of the polarization-dependent signals, which usually causes difficulties in assigning spectral features to specific states. By using polarization-dependent fsTA microscopy, with a probing area smaller than the co-crystal dimensions, CT absorption changes originating from isolated single co-crystals can be determined. Furthermore, most fsTA microscopy experiments employ a single wavelength pump and a single wavelength probe. This has obvious limitations as the full spectral information cannot be probed simultaneously. By combining single wavelength pump with broadband probe beams we were able to measure a spectrum spanning ∼300 nm at different polarizations. Using this technique, the results presented here determine the CT exciton hopping rate constant and show that CT biexciton annihilation to form high-energy free charge carriers occurs in the **PXX**–**Ph4PDI** co-crystal by kinetically outcompeting charge recombination of the individual CT excitons.

## Experimental

### Crystal growth and characterization

Solutions of **PXX** : **Ph4PDI** (1 : 1 molar ratio) were prepared in toluene. Large crystals (>1000 × 10 × 10 μm^3^) were grown by slow vapor diffusion of methanol into the solutions over 1–2 weeks. A suitable crystal was selected and affixed to a loop using Paratone oil on a Bruker APEX-II CCD diffractometer. The crystal was kept at 99.98 K during data collection. Using Olex2,^49^ the structure was solved with the XT structure solution program using intrinsic phasing and refined with the XL refinement package using least squares minimization. The **PXX** : **Ph4PDI** co-crystal structure has been deposited in the Cambridge Crystallographic Data Centre database (CCDC # 1991812). Additional single crystal structural data is reported in the ESI Fig. S1 and Table S1.†

Small crystals (∼50 × 10 × 2 μm^3^) were grown by mixing solutions of **PXX** : **Ph4PDI** (1 : 1 molar ratio) in chlorobenzene and drop-casting onto a clean glass slide. Small crystals were formed on the glass slide after solvent evaporation. The small crystals were used in the optical experiments. For reference, **PXX** and **Ph4PDI** films were grown in separate solutions and deposited using the same technique on different glass slides for analysis using the optical techniques.

### Steady-state absorption and emission microscopy

Steady-state absorption spectra were collected on the small crystals using a xenon arc lamp (Oriel Instruments, model 66902). The beam was polarized using a Glan–Thompson polarizer and then sent into a set of two identical 20× objective lenses focused on the sample. The spot size on the sample was around 5 μm ensuring that each measurement was taken on an isolated cocrystal. The collimated output beam was split using a 10T : 90R beamsplitter. The transmitted beam was focused into a CMOS camera (DCC1645C, Thorlabs) for imaging the sample. The reflected beam was sent to a home-built spectrometer and focused onto a fast line-scan camera (OctoPlus, Teledyne e2v), sampling 130 000 lines per s. First, a reference spectrum was collected with light focused on the bare glass slide, and then the signal (transmission) spectrum was collected on a crystal. Polarization-dependent absorption spectra were collected by rotating the input beam polarization using a broadband half-wave plate (HWP) before focusing it on the sample. To account for spectral deviations of the HWP, the data was background-subtracted using the spectral retardance of the HWP as a guide.

Steady-state fluorescence measurements were obtained using a 0.5 μW, 532 nm excitation beam (Spectra-Physics) on an epi-illumination microscope (Nikon Ti-U) with a 40× magnification objective (Nikon). The captured spectral region between 700–900 nm was smoothed to remove unavoidable strong oscillatory noise between 700–900 nm originating from the CCD detector (PIXIS, Princeton Instruments).

### Transient absorption microscopy

Visible fsTA microscopy was performed using a commercial Yb:KGW, 12 W, 100 kHz repetition rate amplified laser system (Spirit-HE, Spectra-Physics). The 1040 nm fundamental was first decreased to 0.6 W using a variable beamsplitter (BS) and then split using a 50 : 50 BS into two arms. One beam (pump) was sent to a quarter-wave plate, which converted it to a circularly polarized beam, and then into an electro-optic amplitude modulator (EO-AM-NR-C4, Thorlabs), which was phase synchronized to the laser output and modulated the beam at 25 kHz. The modulated output beam was routed through a polarizer. This was followed by second-harmonic generation (SHG) of the 1040 beam to 520 nm using a BBO crystal, blocking the residual fundamental using a short-pass filter (SPF), and spatially filtering the beam. The other 1040 nm beam was sent to a double-pass linear delay line (Newport, IMS600LM), and then focused into an 8 mm thick undoped YAG crystal for white light continuum generation. The beam was then recollimated and the fundamental was removed using a short-pass filter. The pump and probe beams were co-axially combined using a 50 : 50 BS and sent into the same objective lens pair, CMOS camera and spectrometer described above. The polarizations of the pump and the probe beams were varied independently using two HWPs. The pump (probe) energy on the sample was set to 0.4 nJ per pulse (0.1 nJ per pulse) using two neutral density (ND) filters. The pump and probe focused spot sizes (FWHM) on the sample were 2.5 μm and 1.2 μm, respectively (see ESI†), with Gaussian mode shapes. The temporal resolution was <300 fs, as measured by the cross-correlation of the pump and probe on a glass slide using the optical Kerr effect. Due to the coaxial pump/probe geometry, and because the pump and probe spectrally overlap, we were unable to reduce pump scatter on the detector and therefore report fsTA spectra at wavelengths >545 nm.

### Model Hamiltonian for calculating absorption spectra

A model Hamiltonian was used to calculate the polarization-dependent, steady-state absorption spectrum of the co-crystal. The code was written in C and is based on a previously used code for calculating the dynamical and optical properties of singlet fission materials.^50^ The modification of the code included an optically accessible charge-transfer state, the cationic and anionic parts of which were taken to couple to the intramolecular vibrations of the involved donor and acceptor molecules. The Hamiltonian was parameterized based on the experimental steady-state absorption values of the monomeric units and co-crystals, and the TDDFT results for the CT exciton. For details on the form of the Hamiltonian and the parameters used, see the ESI.†

## Results and discussion

### Co-crystal structure

**Fig. 2:**
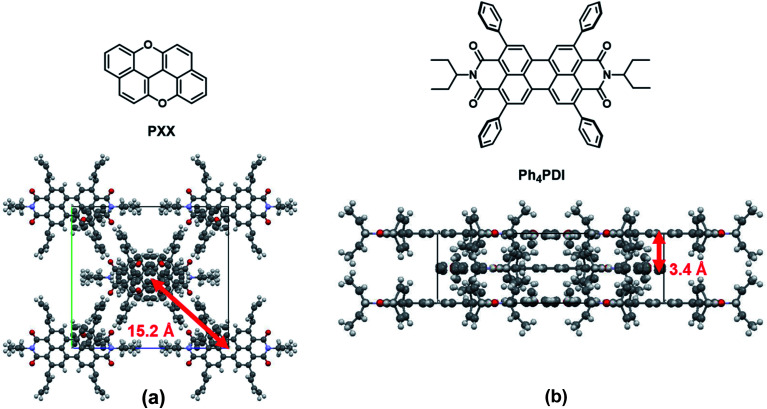
Molecular structures of **PXX** and **Ph4PDI** and views of the crystal structure of the **PXX**–**Ph4PDI** co-crystal along the crystallographic *a*-axis (a) and *b*-axis (b) with annotated inter- and intra-stack distances.

The co-crystal structure, as determined from X-ray diffraction is shown in Fig. 2. The **PXX**–**Ph4PDI** co-crystal is in an orthorhombic *Immm* space group with unit-cell dimensions of *a* = 6.95 Å, *b* = 20.34 Å, *c* = 22.59 Å. There is one **PXX** and one **Ph4PDI** molecule in each unit cell. The **PXX** and **Ph4PDI** molecules π-stack so that their core planes are perfectly parallel with a center-to-center distance of 3.4 Å. The alignment between the two molecules within a unit-cell is such that their S_1_ ← S_0_ transition dipole moments are almost parallel and the line joining their cores is perpendicular to both. Therefore, the transition dipole moment of the CT ← S_0_ transition is perpendicular to the S_1_ ← S_0_ transition dipole moments of both **PXX** and **Ph4PDI.** This is indeed shown to be the case with both steady-state and transient absorption measurements described below. The four phenyl rings on the 2-,5-,8-, and 11-positions of the PDI core have a minor role in determining the HOMO and LUMO energies of this particular PDI derivative^48,51^ and serve mainly as spacers between adjacent **Ph4PDI** molecules, and as interlocks between adjacent π–π stacks. The lateral distance between adjacent π–π stacks is 15.2 Å. The relatively long distance between adjacent stacks means that inter-stack wavefunction overlap is minimal and the interaction between them can be neglected, which permits one to think of the co-crystal as a one-dimensional linear stack of D-A molecules.

### Steady-state absorption and emission spectra

**Fig. 3:**
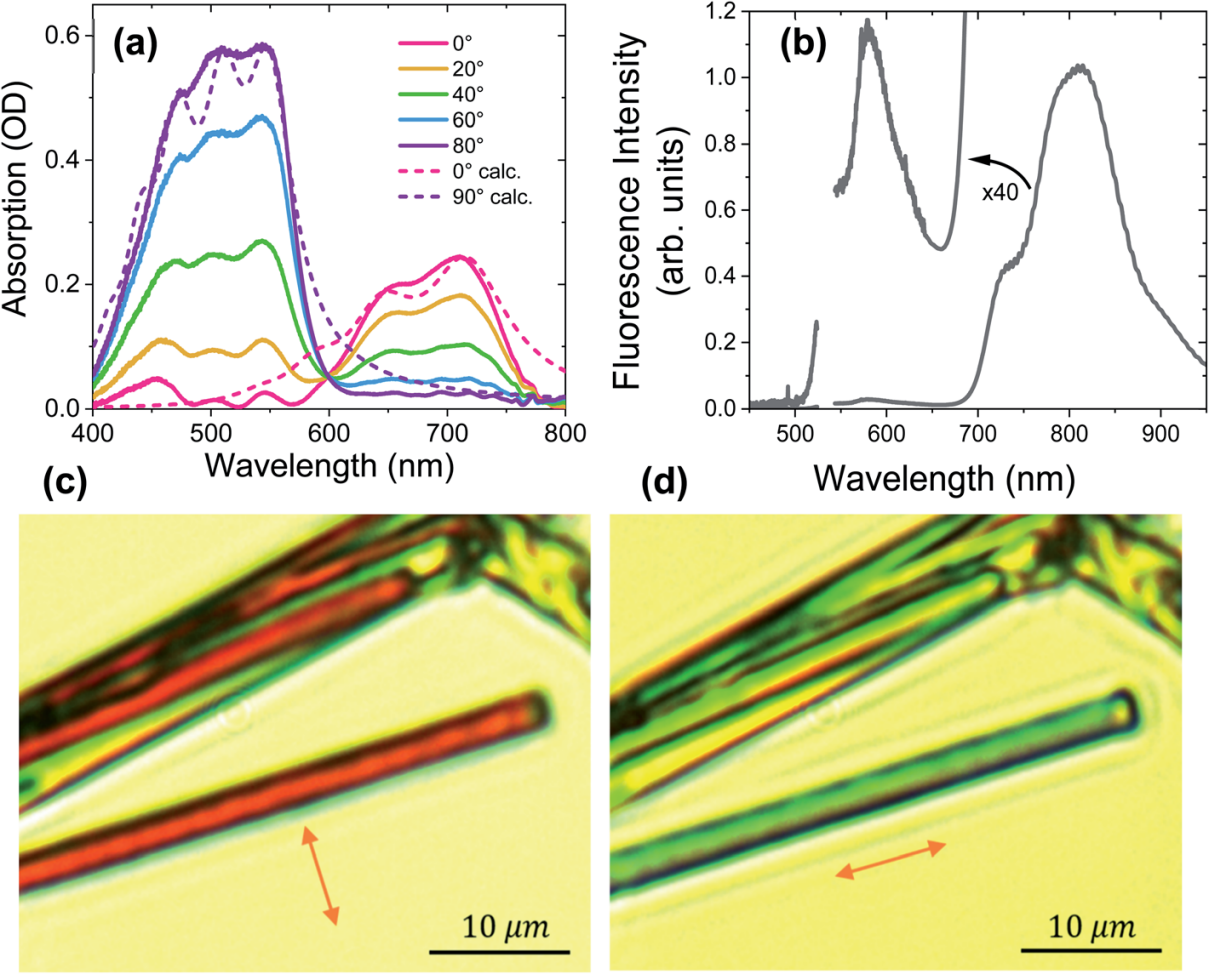
(a) Polarization-dependent steady-state absorption spectra of the **PXX**–**Ph4PDI** co-crystal along with (b) its fluorescence spectrum using 532 nm excitation. The angles are measured relative to the crystal long axis, which is also the π-stacking direction. Dashed lines in (a) are calculated spectra based on the model Hamiltonian described in the text. (c) and (d) Images of co-crystals using polarized white-light illumination. Orange arrows denote the white-light polarization direction.

Fig. 3a shows the polarization-dependent steady-state absorption spectrum of a characteristic **PXX**–**Ph4PDI** co-crystal along with calculated spectra using a model Hamiltonian (see ESI for details†). At 80° polarization relative to the crystal long axis, the crystal absorption spectrum exhibits three peaks at 547, 505, and 454 nm that we assign to the 0–0, 0–1 vibronic bands of **Ph4PDI** and either the 0–2 **Ph4PDI** or the 0–0 **PXX** vibronic bands. It is known that the S_1_ ← S_0_ transition dipole moments of **Ph4PDI**^48^ and **PXX**^52^ lie in the planes of their π systems and are oriented along their long molecular axes. Upon rotating the polarization towards 0° (parallel to the crystal long axis), the monomeric absorption band is strongly attenuated, and the co-crystal spectrum features two new peaks at 650 nm and 710 nm. Importantly, these two new peaks reach their maximum absorption at polarizations where the absorptions of **PXX** and **Ph4PDI** are minimized, and thus are assigned to the CT exciton absorption. The small absorption peaks seen at 0° polarization below 600 nm are due to residual **PXX** and **Ph4PDI** absorption. Examination of the 0–0 and 0–1 vibronic state energy difference of the CT band (Fig. 3a, 80° polarization) yields 0.18 eV, which is in good agreement with the calculated absorption spectrum based on the model Hamiltonian. Since the same vibrational quantum was used to calculate both the **PXX** and **Ph4PDI** S_1_ ← S_0_ transitions and the CT ← S_0_ transition, the good agreement between the experimental and calculated spectra for both polarizations suggests that the CT exciton vibronic coupling originates mainly from vibrational motion within the **PXX** and **Ph4PDI** monomers.

The unpolarized fluorescence spectrum of the co-crystal using 532 nm excitation is shown in Fig. 3b. The spectrum shows two fluorescence bands, a strong one at wavelengths >700 nm and a weak one (see 40× magnification in Fig. 3b) at wavelengths <650 nm. The mirror symmetry of these peaks relative to the absorption peaks permits assignment of the stronger peak to CT exciton fluorescence and the weaker peak to that of **Ph4PDI**. Comparing the lowest energy CT absorption and the highest energy CT emission bands shows that the curves cross at 1.71 eV, which is the CT exciton energy.

For comparison, the absorption spectra of **PXX** and **Ph4PDI** films were also measured (Fig. S2†). The **Ph4PDI** film shows pronounced vibronic features from the 0–0 to 0–3 vibronic bands of **Ph4PDI** at around the same wavelengths as the co-crystal. The fluorescence spectrum obtained of the **Ph4PDI** film shows a mirror image of the absorption spectrum with a Stokes shift of ∼60 nm. The steady-state absorption of a **PXX** film shows a peak at 453 nm, in agreement with literature results.^47,53^ Importantly, **PXX** does not absorb at wavelengths >500 nm, enabling selective excitation of the **Ph4PDI** molecules in the co-crystal.

Fig. 3c and d show images of typical **PXX**–**Ph4PDI** co-crystals taken with the microscope using linearly polarized white light illumination. The arrows denote the polarization direction of the white light. The co-crystals are distinguished by the evident color change upon changing the polarization of the white light. When the illuminating beam is polarized perpendicular to the long crystal axis, the crystals appear as dark red, while for polarization parallel with respect to the crystal long axis, they appear light blue-green, which is consistent with the co-crystal absorption spectra.

### Transient absorption microscopy

**Fig. 4:**
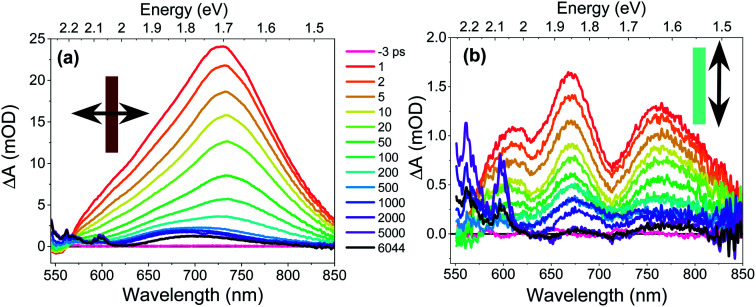
Transient absorption spectra of a **PXX**–**Ph4PDI** co-crystal. Arrows denote probe polarizations relative to the co-crystal long axis (rectangle). The pump–probe delay times refer to both (a) and (b).

The CT exciton dynamics of the **PXX**–**Ph4PDI** co-crystal were investigated using polarization-dependent fsTA microscopy. It was found that the fsTA spectral shape was pump polarization independent, except for affecting the magnitude of Δ*A*, and is consistent with selectively exciting **Ph4PDI**. Conversely, the fsTA spectra were very sensitive to the probe polarization (Fig. 4). Following photoexcitation, when the probe polarization is perpendicular to the crystal long axis (Fig. 4a), a broad, positive absorption peak with a maximum at 732 nm appears within the 300 fs instrument response, which is assigned to formation of **Ph4PDI˙−**^54^ as part of the **PXX˙+**–**Ph4PDI˙−** CT exciton. This peak red shifts slightly to 735 nm in the first few picoseconds, which is most likely a result of a small structural relaxation. At times >500 ps the peak blue shifts to about 700 nm and the shoulder at 550–625 nm sharpens to give bands at 565 and 600 nm, which are assigned to **PXX˙+**.^53^ These bands are blue-shifted somewhat from their absorption maxima in solution most likely because of the low polarity crystalline environment (see below). The apparent lack of a ground state bleach feature at 550–600 nm is most likely a result of its cancellation by the large positive absorption feature extending from 550–850 nm. This absorption lives well beyond the 8 ns maximum pump–probe delay time window of the fsTA microscopy experiment. The fact that the absorption features that live >8 ns have spectra characteristic of **PXX˙+** and **Ph4PDI˙−** suggests that they result from the free charge carriers.

In contrast, when the probe polarization is parallel to the crystal long axis (Fig. 4b) the early-time spectra show two negative features at 630 and 710 nm superimposed on a broad positive background. The positions and relative magnitudes of these negative features are in good agreement with the positions and relative magnitudes of the positive steady-state absorption peaks seen when the light is polarized parallel to the crystal long axis. Therefore, we associate these valleys with the ground-state bleach of the CT absorption. At longer pump–probe delay times, these features decay to give a broad absorption at *λ* > 680 nm, which again is assigned to **Ph4PDI˙−**,^54^ with some distortion from the superimposed ground state bleach along with the appearance of the sharper **PXX˙+** features at 565 and 600 nm.

### CT exciton dynamics

The excitation density of the pump pulses used to excite the co-crystal is 7 × 10^19^ cm^−3^ and the corresponding average fraction of CT excitons produced per number of donor–acceptor pairs in the excitation volume is about 0.13 (see ESI†). Therefore, if the CT excitons are trapped at the sites where they are formed (*k*_hop_ = 0 in Fig. 1) and one assumes a Poisson distribution (see ESI†), then only 1.8% of the CT excitons formed are adjacent to a second CT exciton. This means that at most 1.8% of the CT excitons would annihilate to form free charge carriers. However, the fsTA spectra in Fig. 4 show that Δ*A* at 5 ns is 7% of Δ*A* at 1 ps, implying that CT exciton hopping (or site-to-site tunneling) along the one-dimensional ⋯DADA⋯ π-stacks is leading to a larger CT biexciton annihilation yield than predicted by Poisson statistics for trapped CT excitons.

Kinetic models for exciton hopping and decay on a one-dimensional lattice have been presented previously.^55,56^ These treatments show that at early times, when the exciton concentration is high, exciton hopping and subsequent annihilation can be described by a *t*^−1/2^ time dependence, while at longer times, when the concentration is low, a more complex trap-dependent decay is observed. The spectra shown in Fig. 4 reveal the instrument-limited formation of **PXX˙+**–**Ph4PDI˙−** excitons, so that *k*_CS_ ≥ 3.3 ± 0.1 × 10^12^ s^−1^. The early-time CT exciton dynamics over the time range 0.3 ps < *t* < 500 ps were then modeled as a bimolecular, one-dimensional decay process, captured by the equation:^56^1
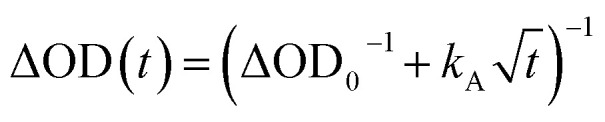
where ΔOD_0_ = ΔOD (*t* = 0) and *k*_A_ is the annihilation rate constant. The exciton hopping rate constant, *k*_hop_, can be obtained from (ref. 56)2
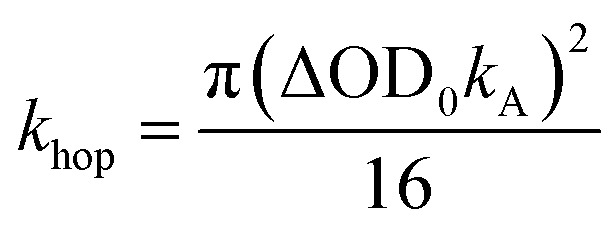


**Fig. 5:**
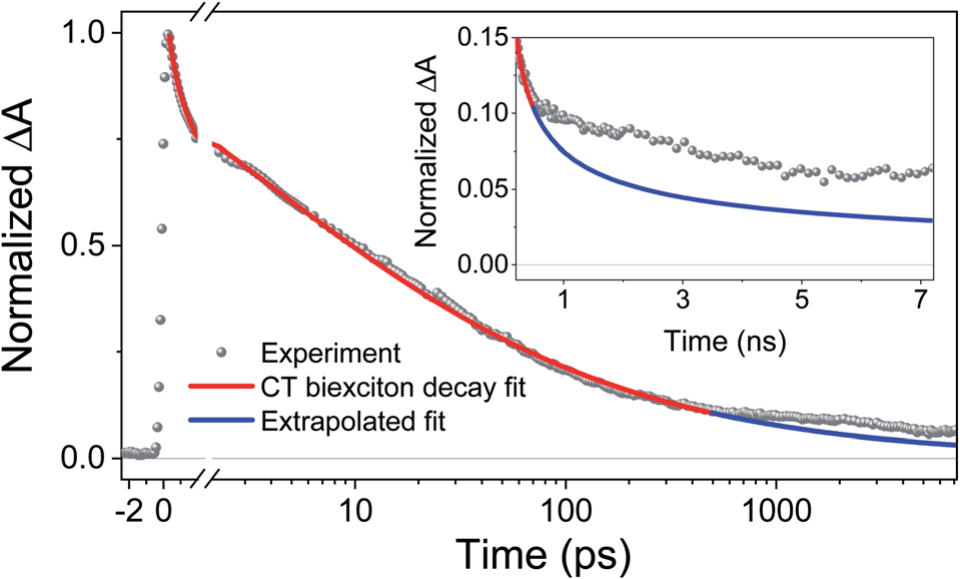
Average of eight normalized kinetic curves in the wavelength range of 665–700 nm. The data over the 0.3 ps < *t* < 500 ps time range were fit to eqn (1), where after normalization ΔOD_0_ was set to 1. Inset: the data for times > 500 ps were not fit. The blue trace is the early time fit extrapolated to longer times to highlight the deviation from *t*^−1/2^ dependence. Inset: an expanded view of the difference between the data and the fit at longer times.

To obtain *k*_hop_, eight kinetic curves in the wavelength range of 665–700 nm were normalized and then averaged to a single kinetic curve. The data over the time range 0.3 ps < *t* < 500 ps were fit to eqn (1), where after normalization ΔOD_0_ was set to 1. The normalized, averaged kinetic curve together with the fit is shown in Fig. 5, which yields *k*_hop_ = 5.2 ± 0.1 × 10^10^ s^−1^. We will discuss the possible physical mechanism of CT exciton hopping below.

It is important to note that this model assumes that the CT excitons decay to ground state at long times. However, it is clearly seen in Fig. 5 that there remains a long-lived component on the experimental curve. The blue trace in Fig. 5 is an extrapolation of the early time fit to 7 ns that highlights the slower decay at longer times. This long-lived species has a fsTA spectrum similar to that of **PXX˙+**–**Ph4PDI˙−** (Fig. 4), which suggests that CT biexciton annihilation leads to D˙^+^–A–D–A˙^−^, producing free charge carriers in the one-dimensional π stacks. Further, the modest blue shift of the transient spectrum for *t* > 500 ps is consistent with free charges that are not significantly Stark-shifted by the presence of a nearby charge. Such long-lived electron–hole pairs have been seen previously by Andersson *et al.* in a D_1_–A_1_–D_2_–A_2_ system in toluene, where photodriven formation of D_1_˙^+^–A_1_˙^−^–D_2_˙^+^–A_2_˙^−^ using two sequential laser pump pulses leads to charge recombination of the central two ions to produce D_1_˙^+^–A_1_–D_2_–A_2_˙^−^, which lives for >650 ns.^46^

Given that triplet excitons having significant CT character have been observed previously in anthracene–PMDA and phenanthrene–PMDA single crystals,^23,27–29^ it is important to consider the alternative possibility that the long-lived species might be a triplet exciton. However, several lines of evidence argue strongly against the long-lived species observed here being a triplet exciton. First, the spectrum of the long-lived species agrees with that of **PXX˙+**–**Ph4PDI˙−**. Second, the triplet states of **Ph4PDI**^57^ and **PXX**^52^ have little or no absorption at *λ* > 650 nm. Third, conventional spin–orbit-induced intersystem crossing (SO-ISC) for both **PXX** and **Ph4PDI** are very slow as evidenced by their high fluorescence quantum yields.^52,58^ Since the formation of the long-lived species occurs in <500 ps, an ultrafast ISC mechanism is needed to produce triplets. However, the π-stacked **PXX˙+**–**Ph4PDI˙−** geometry mitigates against an ultrafast ISC mechanism. Spin–spin exchange coupling in **PXX˙+**–**Ph4PDI˙−** should be very large, precluding radical-pair ISC;^59^ in addition, ultrafast spin–orbit charge-transfer ISC requires the π systems of **PXX** and **Ph4PDI** to be perpendicular.^60^

**Fig. 6:**
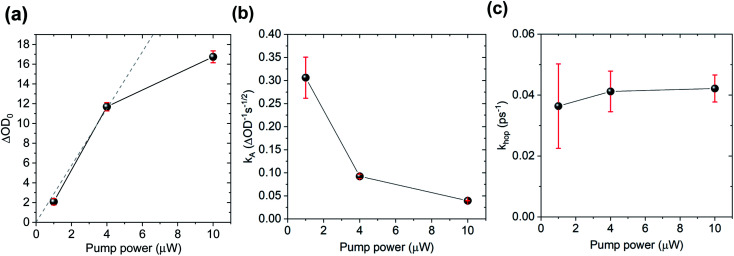
Biexciton annihilation model fitting parameters (a) ΔOD_0_, (b) *k*_A_, and (c) (*k*_hop_) at different pump fluences.

To further test the biexciton annihilation model, we repeated the experiment on a different co-crystal with lower pump fluences. We used a similar fitting procedure for the biexciton annihilation model but did not normalize the kinetic curves to maintain ΔOD_0_ as a fitting parameter in eqn (1). Starting from the origin, at average pump powers of 0–4 μW, ΔOD_0_ follows a linear trend, which suggests that the initial exciton concentration depends linearly on the pump fluence (Fig. 6a). Beyond 4 μW the number of initial CT states tapers off as we enter the non-linear excitation regime. The annihilation rate, *k*_A_, also changes with pump fluence since it depends on the total number of generated CT states (Fig. 6b). However, using eqn (2) to calculate the hopping rate, *k*_hop_, we see that it is constant within error at all pump power levels; thus, supporting the CT exciton migration model.

### CT exciton and biexciton energetics

The annihilation of two CT excitons results in an electron–hole pair in which the charges are initially three times farther apart. The CT exciton state energy can be calculated from the redox potentials of the donor and acceptor, their Coulomb attraction energy and polarization energy of the charges interacting with their surrounding environment using:^61^3
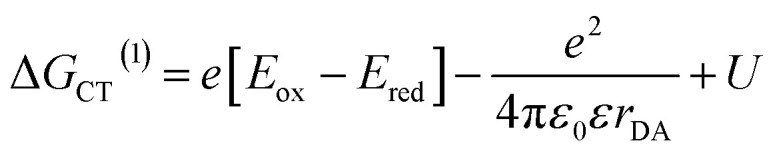
where *e* is the charge of the electron, *E*_ox_ and *E*_red_ are the oxidation and reduction potentials of the donor and acceptor, respectively, *ε*_0_ is the permittivity of free space, *ε* is the static dielectric constant of the medium, and *r*_DA_ is the donor–acceptor distance, respectively, and the term *U* is an environmental correction to the redox potentials, often referred to in the solid state as the polarization energy of the charges on the crystal lattice. Given that *E*_ox_(**PXX**) = 0.76 V^53^ and *E*_red_(**Ph4PDI**) = −0.55 V,^58^ both *vs.* SCE, Δ*G*_CT_^(1)^ = 1.71 eV, as determined from the CT absorption and emission spectra, and using the measured dielectric constant for solid perylene, *ε* = 3.34,^62^ and *r*_DA_ = 3.4 Å obtained here, then *U* = 1.67 eV. We can extend eqn (3) to account for *n* adjacent excitons by replacing the single coulombic term with the pairwise interactions of each of *m* (*m* = 2*n*) charges:4
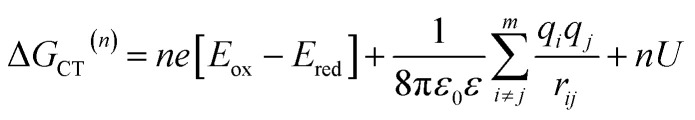
where *r*_*ij*_ is the separation between charges *q*_*i*_ and *q*_*j*_. If *U* = 1.67 eV for each of the two excitons in a uniformly spaced D˙^+^–A˙^−^–D˙^+^–A˙^−^ array, we calculate a biexciton energy of Δ*G*_CT_^(2)^ = 3.00 eV. Following annihilation to produce a single, spatially separated electron–hole pair D˙^+^–A–D–A˙^−^, with *r*_12_ = 10.2 Å, eqn (4) gives the resultant electron–hole pair energy, Δ*G*_eh_ = 2.55 eV. This amounts to a free energy change for CT biexciton annihilation of Δ*G*_A_ = −0.45 eV, assuring that the charge separation process is indeed spontaneous. The spatially separated electron–hole pair also has a 0.84 eV higher energy than the vertical (contact pair) CT exciton. Thus, we expect relaxation of this high energy electron–hole pair to the ground state to be relatively slow as a result of the need to transfer significant energy to crystal lattice phonons, and thus should be in the Marcus inverted region of the rate *vs.* free energy profile.^63,64^

**Fig. 7:**
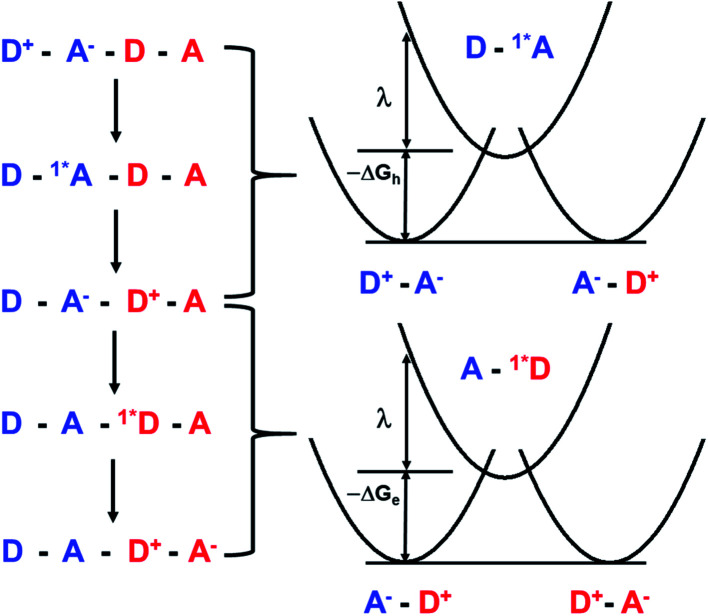
Energy level schematic illustrating the superexchange mechanism for CT exciton tunneling within a ⋯DADA⋯ π-stacked array. The dots indicating the fact that D^+^ and A^−^ are radicals have been removed for clarity.

As noted above, the CT biexciton annihilation yield in the **PXX**–**Ph4PDI** co-crystal exceeds that predicted by the statistics of trapped CT states; thus, it is useful to consider whether the system energetics are consistent with the observed CT exciton hopping rate (*k*_hop_) in the D–A co-crystal. Using the rate *vs.* free energy expression from semiclassical electron transfer theory^65^5
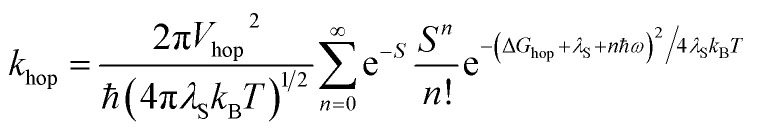
with *k*_hop_ = 5.2 × 10^10^ s^−1^, *ω* = 1500 cm^−1^,^65^*λ*_S_ = 0.2 eV, *T* = 295 K, *S* = *λ*_I_/*ℏω*, *λ*_I_ = 0.2 eV, *k*_B_ is Boltzmann's constant, and assuming that Δ*G*_hop_ = 0 for site-to-site transfer, the electronic coupling matrix element for CT exciton hopping *V*_hop_ = 243 cm^−1^ (see ESI for details†). Movement of CT excitons within ⋯DADA⋯ π-stacked arrays has been suggested to occur *via* a correlated superexchange (tunneling) mechanism (Fig. 7).^24^ This mechanism involves tunneling of the hole on D˙^+^ to the next D as well as tunneling of the electron on A˙^−^ to the next A using the virtual S_1_ states (Frenkel excitons), ^1^*A and ^1^*D, respectively. An alternative superexchange mechanism for CT exciton migration involves A˙^−^ tunneling to the next A using the intervening D˙^−^ as the virtual bridge state, while D˙^+^ tunnels to the next D using the intervening A˙^+^ as the virtual bridge state.^7,8^ We choose to focus on the superexchange mechanism involving the Frenkel excitons in this analysis because the relevant state energies are known from our experimental data. The electronic coupling matrix element for this process, *V*_hop_ = *V*_h_ + *V*_e_, where *V*_h_ and *V*_e_ are the superexchange matrix elements for the hole and electron transfers, respectively. Both *V*_h_ and *V*_e_ can be described by second-order perturbation theory^66–68^ and depend on the product of the initial state – bridge state and bridge state – final state electronic coupling divided by the initial state – bridge state energy gap evaluated at the crossing point of their potential energy curves (Fig. 7), thus6
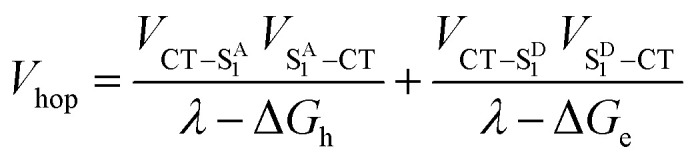


Given that the initial and final states are the same, 
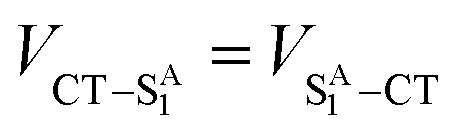
 and 
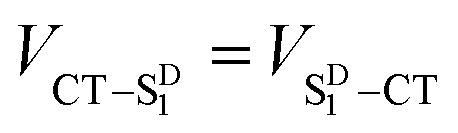
. Moreover, Brédas and co-workers have provided evidence that the electronic coupling matrix elements for hole and electron transfer are comparable for D–A co-crystals that exhibit significant hole and electron mobilities.^7,8^ Thus, eqn (6) can be simplified to7
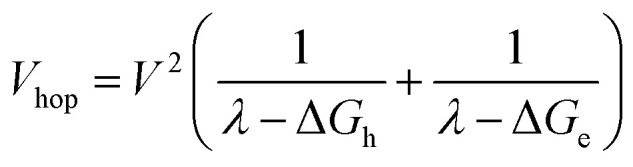


Given that the measured energies of ^1^*Ph4PDI, ^1^*PXX, and the **PXX˙+** –**Ph4PDI˙−** CT exciton in the co-crystal are 2.2, 2.8 and 1.7 eV, respectively, Δ*G*_h_ = −0.5 eV and Δ*G*_e_ = −1.1 eV, and that *λ* = 0.4 eV (see ESI for details†), eqn (7) gives *V* = 1050 cm^−1^ (0.13 eV). Note that this value of *V* is also the electronic coupling matrix element for the initial electron transfer from **PXX** to ^1^*Ph4PDI to give the CT exciton **PXX˙+**–**Ph4PDI˙−**, which suggests that this initial charge separation is most likely adiabatic, consistent with the measured instrument limited rate constant *k*_CS_ ≥ 3.3 × 10^12^ s^−1^ for this process.

## Conclusions

The structurally well-defined **PXX**–**Ph4PDI** D–A co-crystal investigated in this work shows distinct CT absorption and emission spectra. The perpendicular transition moments of the CT exciton and the **PXX** and **Ph4PDI** monomers enable us to isolate and study the CT exciton exclusively without spectral overlap. The CT exciton was analyzed using steady-state absorption and emission microscopy, and broadband fsTA microscopy using polarized pump and probe beams. The CT exciton forms with *k*_CS_ ≥ 3.3 × 10^12^ s^−1^ after selective photoexcitation of **Ph4PDI** and decays to a long-lived species with fsTA spectra similar to **PXX˙+**–**Ph4PDI˙−**. The fsTA data show that the CT excitons decay by one-dimensional CT exciton tunneling in the ⋯DADA⋯ π-stacked arrays followed by CT biexciton annihilation to form high-energy long-lived electron–hole pairs in the co-crystal. These energetic charge carriers may prove useful in applications ranging from photovoltaics and opto-electronics to photocatalysis.

## Conflicts of interest

There are no conflicts to declare.

## Supplementary Material

SC-011-D0SC03301D-s001

SC-011-D0SC03301D-s002
