# Supplementary material for: Charge-transfer biexciton annihilation in a donor–acceptor co-crystal yields high-energy long-lived charge carriers
Source: Chem Sci. 2020 Aug 13;11(35):9532–41. doi: 10.1039/d0sc03301d (PMC8162030; doi:10.1039/d0sc03301d)
Supplement: SC-011-D0SC03301D-s001 [file SC-011-D0SC03301D-s001.pdf]

Electronic Supplementary Material (ESI) for Chemical Science.
This journal is © The Royal Society of Chemistry 2020

Supporting Information for

Charge-Transfer Biexciton Annihilation in a Donor-Acceptor Co-crystal yields High-Energy Long-Lived Charge Carriers

Itai Schlesinger, Natalia E. Powers-Riggs, Jenna L. Logsdon, Yue Qi, Stephen A. Miller, Roel Tempelaar, Ryan M. Young, and Michael R. Wasielewski*

Department of Chemistry and Institute for Sustainability and Energy at Northwestern, Northwestern University, 2145 Sheridan Road, Evanston, Illinois 60208-3113

Contents

1. Single crystal X-ray structure data. 2
2. Crystal structure determination and refinement. 3
3. Additional Steady-state absorption Spectra 4
4. Pump and probe spot sizes 5
5. Excitation density and fraction of molecules excited calculations 6
6. Calculation of the fraction of CT excitons adjacent to one another 7
7. Calculation of reorganization energies and charge transfer rates 9
8. Model Hamiltonian for calculating polarization-dependent steady-state absorption spectra ...10
9. Time-dependent density functional theory 14
10. References 15

1. Single crystal X-ray structure data.

Table S1. Summary of crystal parameters for **PXX:Ph$_4$PDI.**

Identification code	Ph4PDI_PXX
Empirical formula	$C_{78}H_{56}N_2O_6$
Formula weight	1117.24
Temperature/K	100.1
Crystal system	orthorhombic
Space group	Immm
a/Å	6.9513(19)
b/Å	20.343(7)
c/Å	22.590(6)
α/°	90
β/°	90
γ/°	90
Volume/Å^3	3194.4(16)
Z	2
ρ_{calc}g/cm^3	1.162
μ/mm^{-1}	0.577
F(000)	1172.0
Crystal size/mm^3	0.244 × 0.073 × 0.044
Radiation	CuKα (λ = 1.54178)
2Θ range for data collection/°	5.846 to 118.632
Index ranges	$-4 \leq h \leq 7$, $-18 \leq k \leq 20$, $-16 \leq l \leq 25$
Reflections collected	5237
Independent reflections	1273 [R_{int} = 0.0310, R_{sigma} = 0.0300]
Data/restraints/parameters	1273/363/185
Goodness-of-fit on F^2	1.881
Final R indexes [I>=2σ (I)]	R_1 = 0.1087, wR_2 = 0.3982
Final R indexes [all data]	R_1 = 0.1380, wR_2 = 0.4373
Largest diff. peak/hole / e Å^{-3}	0.45/-0.45

2. Crystal structure determination and refinement.

PXX-Ph$_4$PDI crystallized from chloroform and methanol. The structure was solved with XT intrinsic phasing solution package. The structure was refined with XL least squares minimization. **PXX** was imported through the FRAGFEND command and solved isotropically. **PXX** lies on a symmetry operator, so the molecule was set in Part -1 and each position given 0.125 occupation. The **PXX** moiety was constrained with EADP and FLAT commands and Squeeze was used to remove disordered toluene solvent molecules. Dfix 2.1 was applied to C9-C9A, and Dfix 2.4 was applied to C10-C10A. C13 experiences disorder out of the symmetry element it resides on, not modeled here. The structure was deposited in the Cambridge Crystallographic Data Center (CCDC 1991812).

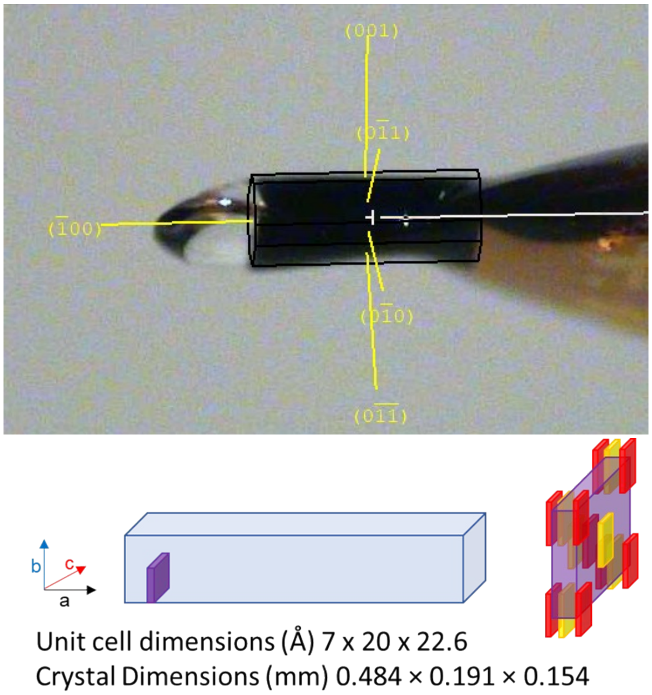

Figure S1. The needle-like crystals have two short axes of similar magnitude and one significantly longer axis. Indexing the crystal faces after determining the unit cell reveals that the small face at the end of the needle corresponds to the (100) plane, indicating the long axis along the macroscopic crystal corresponds to the 7 Å *a* axis of the unit cell, or the direction of π -π stacking.

3. Additional Steady-state absorption Spectra

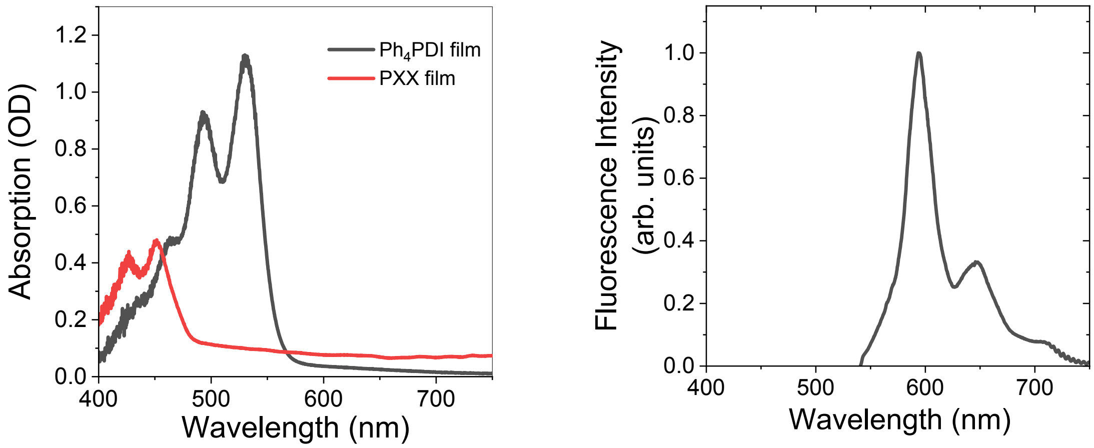

Figure S2. (a) Ground state absorption of ~0.1 μm thick polycrystalline **Ph$_4$PDI** and **PXX** films. (b) Fluorescence emission from the **Ph$_4$PDI** film using 532 nm excitation.

4. Pump and probe spot sizes

The pump and probe beams were independently focused on a glass slide and imaged through the microscope setup on a CMOS camera (see Experimental section). The mode shape and size of each of the spots was extracted using a 2d fit to the Gaussian function

$$z = \exp\left[-\frac{1}{2}\left(\frac{(x - x_c)\cos\theta + (y - y_c)\sin\theta}{w_1}\right)^2 - \frac{1}{2}\left(\frac{-(x - x_c)\sin\theta + (y - y_c)\cos\theta}{w_2}\right)^2 \right]$$

Where $x_c, y_c, w_1, w_2, \theta$ are the center x and center y positions, the widths along the two principal axes and the angle of rotation around the z axis, respectively. The FWHM for each principal axis was calculated using

$$FWHM_i = 2\sqrt{2\ln 2}\, w_i, \quad i = 1,2$$

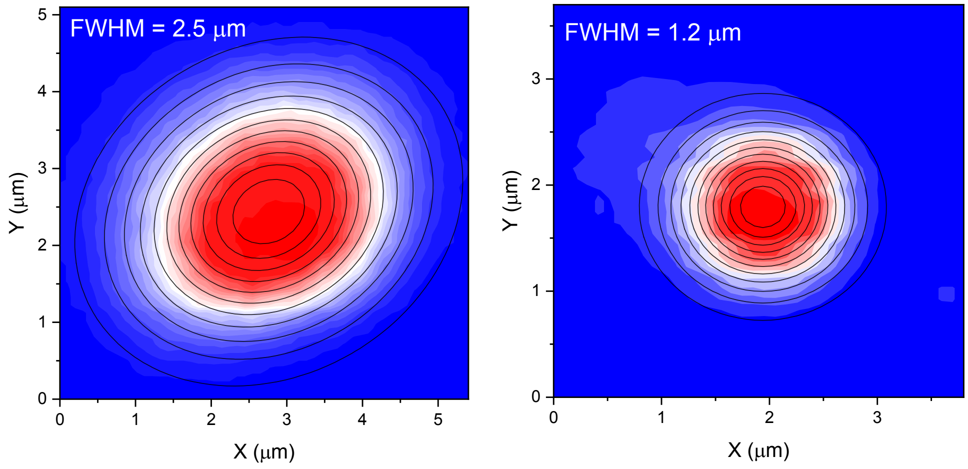

Figure S3. False-color images of the pump (left) and probe (right) spots focused on a glass slide as captured through the TA microscope on a CMOS camera. The black lines are contours of 2d Gaussian fits. The average FWHM of the two principal axes of each fitted Gaussian is listed on the corresponding image.

5. Excitation density and fraction of molecules excited calculations

Table S2. Parameters for excitation density calculation.

λ	P_{avg}	f_{rep}	M	r	t	v
$520\,nm$	$10\,\mu W$	$25\,kHz$	1	$1.5\,\mu m$	$2\,\mu m$	$3.2 \times 10^{-27}\,m^3$

Then number of photons in the excitation volume per pulse is:

$$N_p = \frac{\lambda P_{avg}}{hcf_{rep}}$$

Where $\lambda, P_{avg}, h, c, f_{rep}$ are the wavelength, average pulsed pump power, Planck's constant, the speed of light in vacuum, and the pump repetition rate, respectively. The number of molecules in the excitation volume is

$$N_m = M\frac{V}{v} = M\frac{\pi r^2 t}{v}$$

Where M, V, v, r, t are the number of molecules per unit cell that can absorb a pump photon, excitation volume, unit cell volume, spot radius and crystal thickness.

The excitation density is then

$$\xi = \frac{N_p}{V}$$

so that $\xi = 7 \times 10^{19}$ cm^{-3}. An upper bound on the fraction of donor-acceptor pairs excited in the co-crystal with each laser pulse, $f = N_p/N_m$, can be calculated by assuming every molecule that can absorb a photon does so. The crystal thickness varies the most between crystals but is assumed to be comparable to the crystal width as imaged on the microscope. With these values we get

$$N_p < 1.05 \times 10^9, \quad N_m > 7.85 \times 10^9$$

$$f < 0.13$$

6. Calculation of the fraction of CT excitons adjacent to one another

The initial average fraction of occupied CT sites in the crystal is f, and the distribution of the CT states in the crystal is Poissonian with an average spacing of $1/f$ between CT states. Since the lattice is discrete, the distance between each pair of CT excitons is a random variable, X, which is distributed geometrically with probability parameter f:

$$Pr(X = k) = f(1 - f)^{k - 1}$$

We now examine an arbitrary site in this lattice. Setting A as the event of having the previous site occupied (whether the current site is occupied or not), and B as the event of having the examined site occupied, the probability of having two nearest neighbors CT excitons in the crystal is:

$$P(A \cap B) = P(A | B)P(B)$$

From the previous definitions:

$$P(A | B) = Pr(X = 1) = f$$

and

$$P(B) = f$$

So that $P(A \cap B) = f^2$. For our calculated excitation density of $f \approx 13.3\% = 0.133$, assuming that every excited state Ph_4PDI converts with 100% probability to a CT state, the percentage of adjacent CT states in the crystal is $f^2 \approx 1.8\%$.

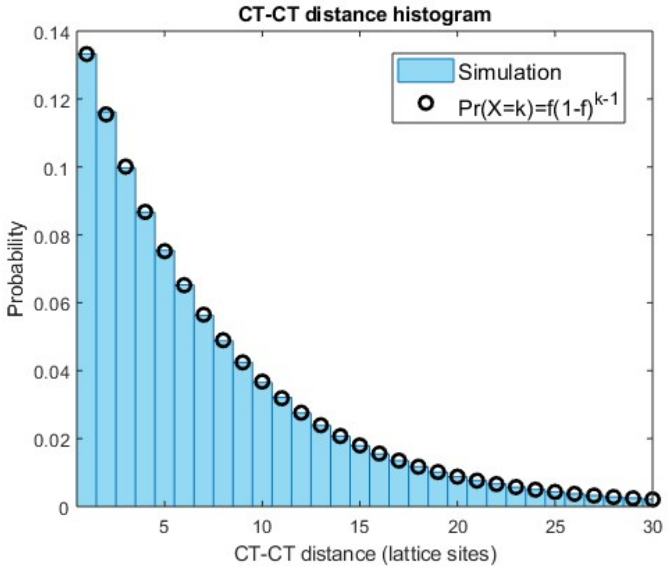

Figure S4. Histogram of CT-CT distances simulated in a 1D lattice with 10^7 sites with site occupation probability of 13.3%. Overlaid in circles is the calculated geometric distribution with $f = 0.133$.

7. Calculation of reorganization energies and charge transfer rates

The internal nuclear reorganization energies, for **PXX** → **PXX**$^{\bullet+}$, λ_I = 0.08 eV, and **Ph$_4$PDI** → **Ph$_4$PDI**$^{\bullet-}$ λ_I = 0.13 eV,[1] were obtained by calculating the geometry-optimized ground state energies of the neutral species, E_0, the ionic species, E', and the energy of the ions at the ground state geometries, E'_0. The calculations were performed with the B3LYP theory level functional using the 6-31G** basis set. All calculations were run in QChem 5.1.[2]

The value of the environmental contribution to the reorganization energy, λ_S, was computed starting with eq 1 from the main text:

$$\Delta G_{CT}^{(1)} = e\left[E_{ox} - E_{red}\right] - \frac{e^2}{4\pi\varepsilon_0\varepsilon r_{DA}} + U$$

Since $\Delta G_{CT}^{(1)}$= 1.71 eV, and $e\left[E_{ox} - E_{red}\right]$ = 1.31 eV, then the remaining terms equal 0.4 eV. These correction terms are identical to those proposed by Weller using the dielectric continuum model, [3] so that

$$\frac{e^2}{4\pi\varepsilon_0\varepsilon}\left(\frac{1}{2r_D} + \frac{1}{2r_A} - \frac{1}{r_{DA}}\right) = 0.4\ eV$$

Thus

$$\left(\frac{1}{2r_D} + \frac{1}{2r_A} - \frac{1}{r_{DA}}\right) = 0.093\ \text{Å}^{-1}$$

The Marcus expression that also uses dielectric continuum theory to evaluate λ_S is[4-5]

$$\lambda_S = \frac{e^2}{4\pi\varepsilon_0\varepsilon}\left(\frac{1}{2r_D} + \frac{1}{2r_A} - \frac{1}{r_{DA}}\right)\left(\frac{1}{E_{op}} - \frac{1}{E}\right)$$

So that using the above equation with ε_{op} = 2.2 for polarizable aromatic molecules, ε = 3.34 obtained from solid perylene,[6] **Ph$_4$PDI**$^{\bullet-}$, λ_S = 0.19 eV. The total reorganization energy for **PXX-Ph$_4$PDI** → **PXX**$^{\bullet+}$**- Ph$_4$PDI**$^{\bullet-}$ $\lambda = \lambda_I + \lambda_S$ = 0.08 eV + 0.13 eV + 0.19 eV = 0.40 eV.

8. Model Hamiltonian for calculating polarization-dependent steady-state absorption spectra

The electronic Hamiltonian reads:

$$H_{el} = E_D \sum_{n \in D} d_n^{\dagger} c_n^{\dagger} c_n d_n + E_A \sum_{n \in A} d_n^{\dagger} c_n^{\dagger} c_n d_n + E_{CT} \sum_{n \in D} \sum_{m = n \pm 1} d_n^{\dagger} c_m^{\dagger} c_m d_n + t_{LL} \sum_{n} c_n^{\dagger} c_{n \pm 1}$$

Here, the summations run over donor (D) and acceptor (A) molecules, E_D and E_A are the donor and acceptor $S_1 \leftarrow S_0$ transition energies, respectively, E_{CT} is the nearest-neighbor CT energy relative to S_0, and t_{HH} and t_{LL} are the HOMO-HOMO and LUMO-LUMO electron transfer integrals between neighboring molecules. These integrals are identical for each neighboring pair, owing to symmetry. The operators $c_n^{(\dagger)}$ and $d_n^{(\dagger)}$ correspond to the creation/annihilation of an electron in the LUMO and a hole in the HOMO of molecule n, respectively. As the $S_1 \leftarrow S_0$ transitions at the donor and acceptor molecules are highly non-resonant, the dipole-dipole interactions between them is considered to be negligible compared to the electron transfer integrals. We likewise neglect such interactions between equivalent molecules in view of strong screening by the inequivalent bridging molecule.

The total Hamiltonian is:

$$H = H_{el} + \omega \sum_{n} b_n^{\dagger} b_n + \omega \sum_{n \in D,A} \left[\lambda_{D,A} \left(b_n^{\dagger} + b_n \right) + \lambda_{D,A}^2 \right] d_n^{\dagger} c_n^{\dagger} c_n d_n + \omega \sum_{n \in D [illegible]} d_n^{\dagger} c_m^{\dagger} c_m d_n$$

Here, $\lambda_D^2, \lambda_A^2, \lambda_+^2$, and λ_-^2 are the Huang-Rhys (HR) factors for the donor, acceptor, cation and anion, respectively, and $b_n^{(\dagger)}$ represent the ladder operators for an intramolecular vibration with a quantum ω at molecule n ($\hbar = 1$ is taken).

The basis states are

$$|(S_1)_n, \nu\rangle$$

$$|(+)_n, \nu_+; (-)_{n\pm1}, \nu_-\rangle, n \in D$$

Here ν, ν_+, and ν_- label the number of vibrations in the S_1, cationic, and anionic potentials, respectively.

The transition dipole moment operator is

$$\vec{M} = \vec{\mu}_D \sum_{n \in D} c_n d_n + \vec{\mu}_A \sum_{n \in A} c_n d_n \pm \vec{\mu}_{CT} \sum_{n \in D} \sum_{m = n \pm 1} c_m d_n + H.c.$$

The absorption spectrum in the j-polarized direction is then calculated as

$$A_j(\omega) = \sum_{\alpha} |\langle 0 | M_j | \alpha \rangle|^2 W_j(\omega_\alpha - \omega)$$

Where $|\alpha\rangle$ and ω_α are the eigenstates and energies obtained by solving the time-independent Schrödinger equation, $H|\alpha\rangle = \omega_\alpha|\alpha\rangle$, $|0\rangle$ is the vacuum state where all molecules are in their S_0 level without vibrations, and $W_j(\omega)$ is the lineshape function.

The parameters used to calculate the polarized absorption spectra in Figure 3a in the main text are summarized in Table S3. The vibrational quantum and donor and acceptor HR factors were taken from the energy difference and relative intensities of the vibronic progression observed in the monomer linear absorption spectra (yielding roughly equal parameters for donor and acceptor). The cationic and anionic HR factors were taken to be equal and taken so as to reproduce the CT band in the co-crystal absorption spectrum. Note that all vibrational parameters are in good

agreement with those adopted in detailed modeling of Ph_4PDI reported previously, although the absence of spectral overlap and optical brightness of the CT band renders the present determination of these parameters less ambiguous.[7] A maximum number of vibrational quanta of 3 was taken, where it should be noted that vibrational states were always expressed in their respective eigenbasis (corresponding to S_0, S_1, and CT electronic states).

Values for the donor and acceptor $S_1 \leftarrow S_0$ transition energies as well as the relative CT energy were obtained by aligning the associated bands appearing in the spectral simulations with those observed experimentally, and qualitatively match those obtained in our TD-DFT calculations (Figure 6 in the main text). For simplicity, the HOMO-HOMO and LUMO-LUMO overlap factors were assumed to have equal magnitude, and their value was inferred from the modified vibronic progression observed in the linear absorption spectrum of the co-crystal. The (relative) signs of these overlap factors have very little impact on the spectral properties. Owing to symmetry considerations, they only affect the phase mixing of donor and acceptor singlet states, the degree of which is small as these states are separated by 400 meV. We therefore take the overlap factors to have equal signs. The dipole moments were aligned in accordance with our TD-DFT calculations, and their magnitudes were scaled to match the intensities of the three absorption bands observed experimentally. For the lineshape function, a Lorentzian was taken with a linewidth of 100 meV. Periodic boundary conditions were applied, in order to mimic extended co-crystal sizes.

Table S3. Parameters used to calculate the absorption spectra in Figure 3a in the main text.

E_D	2650 meV
E_A	2260 meV
E_{CT}	1785 meV
t_{HH}	37 meV
t_{LL}	37 meV
$\lvert\vec{\mu}_D\rvert$	0.93 a.u.
$\lvert\vec{\mu}_A\rvert$	0.58 a.u.
$\lvert\vec{\mu}_{CT}\rvert$	0.47 a.u.
ω	175 meV
$\lambda_{D,A}^2$	0.77
$\lambda_{+,-}^2$	0.4
Max. number of vibrational quanta	3
Number of D-A unit cells	5

9. Time-dependent density functional theory

Single-point calculations of ground and excited states for isolated **PXX** and **Ph_4PDI** molecules, as well as a single cofacially stacked **PXX-Ph_4PDI** pair were conducted using time-dependent density functional theory (TDDFT). To reduce computational cost, the two 3-pentyl groups on **Ph_4PDI** were replaced by hydrogen atoms. This is known to have minimal effect on wavefunction and energy calculations.[8] The spacing between the individual monomers in the D-A pair was set to 3.4 Å in accordance with the measured spacing from x-ray diffraction. The calculations were performed with the B3LYP theory level functional using the cc-pVDZ basis set. All calculations were run in QChem 5.1.[2]

Figure S5 depicts an energy level diagram of the isolated **PXX** and **Ph_4PDI** singlets and a **PXX-Ph_4PDI** pair singlet. All energies shown are relative to the calculated **PXX** HOMO. The calculated absorption peaks are blue-shifted relative to the experimental absorption peaks (Figure 3a) due to stronger intermolecular interactions in the condensed environment (film/crystal) relative to vacuum. Interactions between similar molecules (D-D or A-A) in the co-crystal are further suppressed due to a large separation between them, a bridging (D or A, respectively) molecule, and the dielectric environment which acts to screen such interactions and lets us approximate the co-crystal energy levels with the ones calculated for the D-A pair.

For such weakly interacting chromophores, the HOMO and LUMO of the individual chromophores should not change appreciably in a D-A pair. As seen in Figure 7, this is indeed the case, and the calculated values closely match those found in the literature.[9]

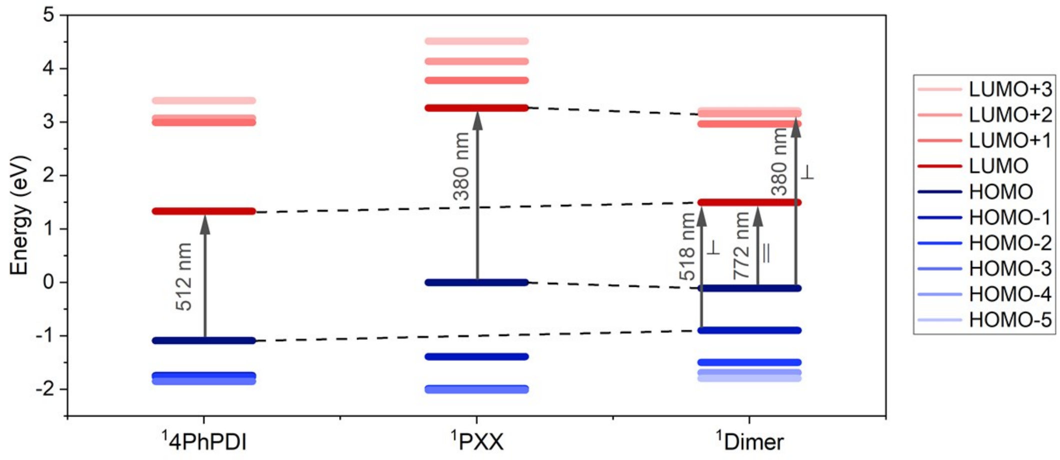

Figure S5. Singlet energy level diagrams of isolated **Ph_4PDI** molecule, isolated **PXX** molecule and **PXX-Ph_4PDI** pair. Arrows represent transitions with appreciable oscillator strength. Perpendicular and parallel symbols mark transition dipole moment direction relative to the line connecting the donor-acceptor cores.

Upon dimerization a new absorption band emerges with non-negligible oscillator strength, which is the HOMO to LUMO excitation. Inspection of the calculated transition dipole moment of this band shows that it points along the line connecting the donor to the acceptor, showing that it is a CT transition. The calculated wavelength for this transition is 772 nm, which is close, but red-shifted relative to the position of the experimental CT band seen in Figure 3.

Examining the energy levels of the D-A pair, we see that there is another good candidate transition to generate a CT state, namely from the D-A pair HOMO, localized on the **PXX**, to the D-A pair LUMO+1, with contribution mainly from the LUMO+1 level of the isolated **Ph_4PDI**. Nevertheless, it was found in our calculations that this transition carries negligible oscillator strength and is therefore not shown in Figure S5.

10. References

1. Hartnett, P. E.; Mauck, C. M.; Harris, M. A.; Young, R. M.; Wu, Y.-L.; Marks, T. J.; Wasielewski, M. R., Influence of anion delocalization on electron transfer in a covalent porphyrin donor-perylenediimide dimer acceptor system. *J. Am. Chem. Soc.* **2017,** *139*, 749-756.

2. Shao, Y.; Gan, Z.; Epifanovsky, E.; Gilbert, A. T. B.; Wormit, M.; Kussmann, J.; Lange, A. W.; Behn, A.; Deng, J.; Feng, X.; Ghosh, D.; Goldey, M.; Horn, P. R.; Jacobson, L. D.; Kaliman, I.; Khaliullin, R. Z.; Kuś, T.; Landau, A.; Liu, J.; Proynov, E. I.; Rhee, Y. M.; Richard, R. M.;

Rohrdanz, M. A.; Steele, R. P.; Sundstrom, E. J.; Woodcock, H. L.; Zimmerman, P. M.; Zuev, D.; Albrecht, B.; Alguire, E.; Austin, B.; Beran, G. J. O.; Bernard, Y. A.; Berquist, E.; Brandhorst, K.; Bravaya, K. B.; Brown, S. T.; Casanova, D.; Chang, C.-M.; Chen, Y.; Chien, S. H.; Closser, K. D.; Crittenden, D. L.; Diedenhofen, M.; DiStasio, R. A.; Do, H.; Dutoi, A. D.; Edgar, R. G.; Fatehi, S.; Fusti-Molnar, L.; Ghysels, A.; Golubeva-Zadorozhnaya, A.; Gomes, J.; Hanson-Heine, M. W. D.; Harbach, P. H. P.; Hauser, A. W.; Hohenstein, E. G.; Holden, Z. C.; Jagau, T.-C.; Ji, H.; Kaduk, B.; Khistyaev, K.; Kim, J.; Kim, J.; King, R. A.; Klunzinger, P.; Kosenkov, D.; Kowalczyk, T.; Krauter, C. M.; Lao, K. U.; Laurent, A. D.; Lawler, K. V.; Levchenko, S. V.; Lin, C. Y.; Liu, F.; Livshits, E.; Lochan, R. C.; Luenser, A.; Manohar, P.; Manzer, S. F.; Mao, S.-P.; Mardirossian, N.; Marenich, A. V.; Maurer, S. A.; Mayhall, N. J.; Neuscamman, E.; Oana, C. M.; Olivares-Amaya, R.; O'Neill, D. P.; Parkhill, J. A.; Perrine, T. M.; Peverati, R.; Prociuk, A.; Rehn, D. R.; Rosta, E.; Russ, N. J.; Sharada, S. M.; Sharma, S.; Small, D. W.; Sodt, A.; Stein, T.; Stück, D.; Su, Y.-C.; Thom, A. J. W.; Tsuchimochi, T.; Vanovschi, V.; Vogt, L.; Vydrov, O.; Wang, T.; Watson, M. A.; Wenzel, J.; White, A.; Williams, C. F.; Yang, J.; Yeganeh, S.; Yost, S. R.; You, Z.-Q.; Zhang, I. Y.; Zhang, X.; Zhao, Y.; Brooks, B. R.; Chan, G. K. L.; Chipman, D. M.; Cramer, C. J.; Goddard, W. A.; Gordon, M. S.; Hehre, W. J.; Klamt, A.; Schaefer, H. F.; Schmidt, M. W.; Sherrill, C. D.; Truhlar, D. G.; Warshel, A.; Xu, X.; Aspuru-Guzik, A.; Baer, R.; Bell, A. T.; Besley, N. A.; Chai, J.-D.; Dreuw, A.; Dunietz, B. D.; Furlani, T. R.; Gwaltney, S. R.; Hsu, C.-P.; Jung, Y.; Kong, J.; Lambrecht, D. S.; Liang, W.; Ochsenfeld, C.; Rassolov, V. A.; Slipchenko, L. V.; Subotnik, J. E.; Van Voorhis, T.; Herbert, J. M.; Krylov, A. I.; Gill, P. M. W.; Head-Gordon, M., Advances in molecular quantum chemistry contained in the q-chem 4 program package. *Mol. Phys.* **2015,** *113*, 184-215.

3. Weller, A., Photoinduced electron transfer in solution: Exciplex and radical ion pair formation free enthalpies and their solvent dependence. *Z. Phys. Chem.* **1982,** *133*, 93-99.

4. Marcus, R. A., On the theory of oxidation-reduction reactions involving electron transfer. I. *J. Chem. Phys.* **1956,** *24*, 966-978.

5. Marcus, R. A., Theory of electron-transfer reactions. VI. Unified treatment for homogeneous and electrode reactions. *J. Chem. Phys.* **1965,** *43*, 679-701.

6. Ishii, K.; Kinoshita, M.; Kuroda, H., Dielectric constant measurement on organic crystalline powder. *Bull. Chem. Soc. Jpn.* **1973,** *46*, 3385-3391.

7. Oleson, A.; Zhu, T.; Dunn, I. S.; Bialas, D.; Bai, Y.; Zhang, W.; Dai, M.; Reichman, D. R.; Tempelaar, R.; Huang, L.; Spano, F. C., Perylene diimide-based Hj- and hJ-aggregates: The prospect of exciton band shape engineering in organic materials. *J. Phys. Chem. C* **2019,** *123*, 20567-20578.

8. Delgado, M. C. R.; Kim, E.-G.; Filho, D. A. d. S.; Bredas, J.-L., Tuning the charge-transport parameters of perylene diimide single crystals via end and/or core functionalization: A density functional theory investigation. *J. Am. Chem. Soc.* **2010,** *132*, 3375-3387.

9. Nakazono, S.; Easwaramoorthi, S.; Kim, D.; Shinokubo, H.; Osuka, A., Synthesis of arylated perylene bisimides through C-H bond cleavage under ruthenium catalysis. *Org. Lett.* **2009,** *11*, 5426-5429.
